# Angiography-derived versus pressure-wire–based physiological assessment to guide percutaneous coronary intervention: a systematic review and meta-analysis of randomised controlled trials

**DOI:** 10.1186/s12872-026-06305-7

**Published:** 2026-07-18

**Authors:** Mahwish Sarwar, Shaikh Muhammad Daniyal, Shaheer Bin Shafiq, Naveen Murad Khatoon, Amna Noor, Muhammad Tahir, Minahil Riaz, Ayan Khalid, Fizza Batool, Minaam Farooq, Augustine George, Romal Jabarkhil

**Affiliations:** 1 Department of Medicine, Dow University of Health Sciences, Karachi, Pakistan; 2 Department of Cardiology, South Texas Health System, McAllen, TX USA; 3 Department of Medicine, Ningarhar Regional Hospital, Jalalabad, Afghanistan

**Keywords:** Angiography-derived fractional flow reserve (FFR), Pressure-wire–based physiology, Percutaneous coronary intervention (PCI), Meta-analysis

## Abstract

**Introduction:**

Angiography-derived fractional flow reserve (FFR) has emerged as a wire-free alternative to pressure-wire–based physiological assessment for guiding percutaneous coronary intervention (PCI). However, comparative clinical outcomes and procedural implications remain uncertain.

**Methods:**

A systematic review and meta-analysis of randomised controlled trials (RCTs) was conducted in accordance with PRISMA guidelines. Databases were searched from inception to April 2026. Studies comparing angiography-derived FFR with pressure-wire–based strategies in patients undergoing PCI were included. Risk ratios (RRs) and mean differences (MDs) with 95% confidence intervals (CIs) were pooled using a random-effects model. Pre-specified subgroup analyses were performed based on technology type and comparator composition.

**Results:**

Three RCTs were included. Angiography-derived strategies demonstrated no significant difference compared with wire-based approaches for the primary composite outcome (RR: 1.14; 95% CI: 0.85 to 1.55), all-cause mortality, cardiac mortality, myocardial infarction (MI), or revascularization. However, angiography-derived guidance significantly reduced procedural time (MD: − 2.83 min; 95% CI: − 4.94 to − 0.72), fluoroscopy time (MD: − 2.50 min; 95% CI: − 3.38 to − 1.61), and contrast volume (MD: − 6.99 mL; 95% CI: − 11.57 to − 2.40).

**Conclusions:**

Angiography-derived physiological assessment provides comparable 1-year clinical outcomes to pressure-wire–based strategies while improving procedural efficiency. However, outcomes may vary by platform, highlighting the importance of technology-specific evaluation in future research.

**Supplementary Information:**

The online version contains supplementary material available at https://doi.org/10.1186/s12872-026-06305-7.

## Introduction

Ischemic heart disease (IHD) remains the leading cause of mortality worldwide, responsible for over 9 million deaths annually and imposing a substantial burden on healthcare systems [1]. Percutaneous coronary intervention (PCI) is a cornerstone therapy for IHD, with millions of procedures performed each year. Yet identifying which stenoses are ischemia-producing remains a central clinical challenge, as visual angiographic interpretation correlates poorly with hemodynamic significance in lesions of intermediate severity [2]. 

Pressure-wire-derived fractional flow reserve (FFR) is the established reference standard for coronary physiological assessment. Randomised trials have shown improved clinical outcomes over angiography-guided and medical-therapy strategies [2, 3], and FFR-guided revascularization holds Class I recommendations in European and North American guidelines [4, 5]. Despite these endorsements, utilisation remains limited by pressure-wire instrumentation, hyperaemic agents, added cost and procedural time, with fewer than one in five intermediate lesions assessed invasively in contemporary registries [6]. 

Angiography-derived FFR has emerged as a wire-free alternative that computes lesion-specific physiology from routine angiographic images [7]. Available systems are mechanistically distinct: patient-specific aortic pressure calibration (FFRangio, vessel FFR [vFFR]) versus fixed-flow assumptions without invasive calibration (quantitative flow ratio [QFR]), with potential implications for diagnostic accuracy. However, their agreement with invasive FFR remains imperfect, and head-to-head outcome data against wire-based strategies have, until recently, been scarce.

Despite the growing availability of angiography-derived FFR, direct synthesis of its clinical and procedural performance against wire-based assessment remains limited. Moreover, whether outcomes differ by the mechanistic class of the angiography-derived system or by comparator composition, FFR alone versus FFR plus non-hyperaemic pressure ratios (NHPR) has not been systematically examined. Three international randomised trials, FAVOR III Europe, FAST III, and ALL-RISE, have directly compared the strategies with discordant results [8–10]. By integrating this evidence and performing pre-specified subgroup analyses across both axes, the present meta-analysis evaluates the clinical efficacy and procedural efficiency of angiography-derived versus pressure-wire-based physiological assessment for guiding coronary revascularization.

## Methods

### Study design and registration

This systematic review and meta-analysis were conducted in accordance with the Preferred Reporting Items for Systematic Reviews and Meta-Analyses (PRISMA) guidelines [11]. The study protocol was prospectively registered in the PROSPERO international register of systematic reviews (*CRD420261367467*).

### Search strategy

A systematic search of PubMed (MEDLINE), Google Scholar, ScienceDirect, ClinicalTrialsFi.gov, and the Cochrane Central Register of Controlled Trials (CENTRAL) comparing angiography-derived FFR with pressure-wire–based physiological assessment in patients undergoing coronary angiography and PCI was performed. The search strategy combined MeSH terms and free-text keywords including: “fractional flow reserve,” “angiography-derived FFR,” “FFRangio,” “vFFR,” “quantitative flow ratio,” “pressure wire,” “coronary physiology,” “percutaneous coronary intervention,” “coronary revascularisation,” and “intermediate coronary stenosis.” Reference lists of relevant review articles and published meta-analyses were additionally hand-searched to capture any studies not retrieved through database searches. All databases were searched from inception through 1st April 2026. The complete search strings for each database and reasons for exclusion of articles are provided in Supplementary Table S1 and Supplementary Table S2 respectively.

### Study selection

Retrieved citations were imported into Rayyan.ai for duplication and screening [12]. Two reviewers (A.N. and N.M.K) independently evaluated titles and abstracts against predefined eligibility criteria, followed by full-text review of potentially eligible records. Discrepancies were resolved by discussion and consensus, with a third reviewer (M.R.) adjudicating where agreement could not be reached. Studies were eligible for inclusion if they: (a) were randomised controlled trials (RCTs); (b) enrolled adult patients aged ≥ 18 years with intermediate coronary artery stenosis undergoing invasive coronary angiography; (c) compared an angiography-derived FFR strategy against a pressure-wire–based physiological assessment strategy to guide coronary revascularization; and (d) reported at least one prespecified clinical outcome of interest at one year of follow-up. Studies were excluded if they: (a) were single-arm studies, observational registries, case series, or conference abstracts without accompanying full-text publications; (b) used angiographic guidance alone (without any physiological assessment) as the comparator; or (c) were published in a language other than English.

### Data extraction

Two reviewers (S.M.D and M.S) independently extracted data. Primary outcomes extracted included the primary composite, all-cause mortality, cardiac mortality, any myocardial infarction (MI), and revascularization. Secondary outcomes included total procedural time, total fluoroscopy time, and volume of contrast media. Discrepancies in extracted data were resolved through discussion and consensus. As the revascularization component of the composite primary endpoint varied slightly across trials, restricted to unplanned, clinically indicated procedures in two trials, but inclusive of any revascularization in one trial, all outcomes were extracted and pooled according to each trial’s original pre-specified definition. The individual trial definition of the primary composite endpoint is summarised in Supplementary Table S3.

### Quality assessment

The methodological quality of included RCTs was independently evaluated by two reviewers (F.B and M.S) using the revised Cochrane Risk of Bias tool for randomised trials (RoB 2.0) [13]. Disagreements were resolved by a third reviewer (S.M.D).

### Certainty of evidence

The certainty of evidence for each outcome was appraised using the Grading of Recommendations, Assessment, Development and Evaluations (GRADE) framework. Evidence was assessed across five domains: risk of bias, inconsistency, indirectness, imprecision, and publication bias, and rated as high, moderate, low, or very low [14]. A Summary of Findings table was prepared using GRADEpro GDT software (Supplementary Table S4).

### Statistical analysis

All meta-analyses were performed using the *meta* package in R (v4.4.2). For dichotomous outcomes, including the primary composite endpoint, all-cause mortality, cardiac mortality, any MI, and revascularization risk ratios (RRs) with 95% confidence intervals (CIs) were calculated. Mean differences (MDs) with 95% CIs were calculated for continuous procedural outcomes, including total procedural time, total fluoroscopy time, and volume of contrast media used. A random-effects model with restricted maximum likelihood (REML) estimation was applied for all pooled analyses to account for anticipated between-study heterogeneity. Statistical heterogeneity was evaluated using the Chi-square test and quantified using Higgins’ I² statistic, categorized as low (< 25%), moderate (25–75%), or high (> 75%). Publication bias was evaluated using a DOI plot and the Luis Furuya-Kanamori (LFK) index, implemented in R version 4.5.1. (S1-S8)

Two pre-specified subgroup analyses were performed. First, a subgroup analysis according to the hemodynamic assumption methodology of each angiography-derived FFR system was conducted. Systems incorporating patient-specific measured aortic pressure as a real-time calibration input during angiography (FFRangio, CathWorks; vFFR, Pie Medical Imaging) were classified as “patient-specific hemodynamic systems”. Systems employing population-averaged, fixed-flow assumptions without invasive pressure calibration (QFR, Medis Medical Imaging) were classified as fixed-flow model-based systems. This classification was pre-specified based on established mechanistic differences in computational methodology and their theoretical implications for individual patient hemodynamic accuracy. The second subgroup was performed according to the composition of the pressure-wire–based comparator arm in each included trial. Trials employing exclusively pressure-wire–derived FFR with mandatory adenosine-induced hyperemia as the sole comparator were classified as FFR-only comparator trials. Trials incorporating a mixed comparator strategy permitting both pressure-wire–derived FFR and NHPR, including instantaneous wave-free ratio and related resting indices, at the discretion of the treating operator, were classified as FFR+NHPR comparator trials. This classification was pre-specified based on the recognized physiological distinction between hyperaemic and non-hyperaemic pressure-wire indices and their potentially differential performance as reference standards for angiography-derived FFR validation. Sensitivity analysis using a leave-one-out approach was performed for each outcome to assess the robustness and stability of the pooled estimates. A two-sided P-value of < 0.05 was considered statistically significant throughout.

## Results

### Study selection

**Fig. 1:**
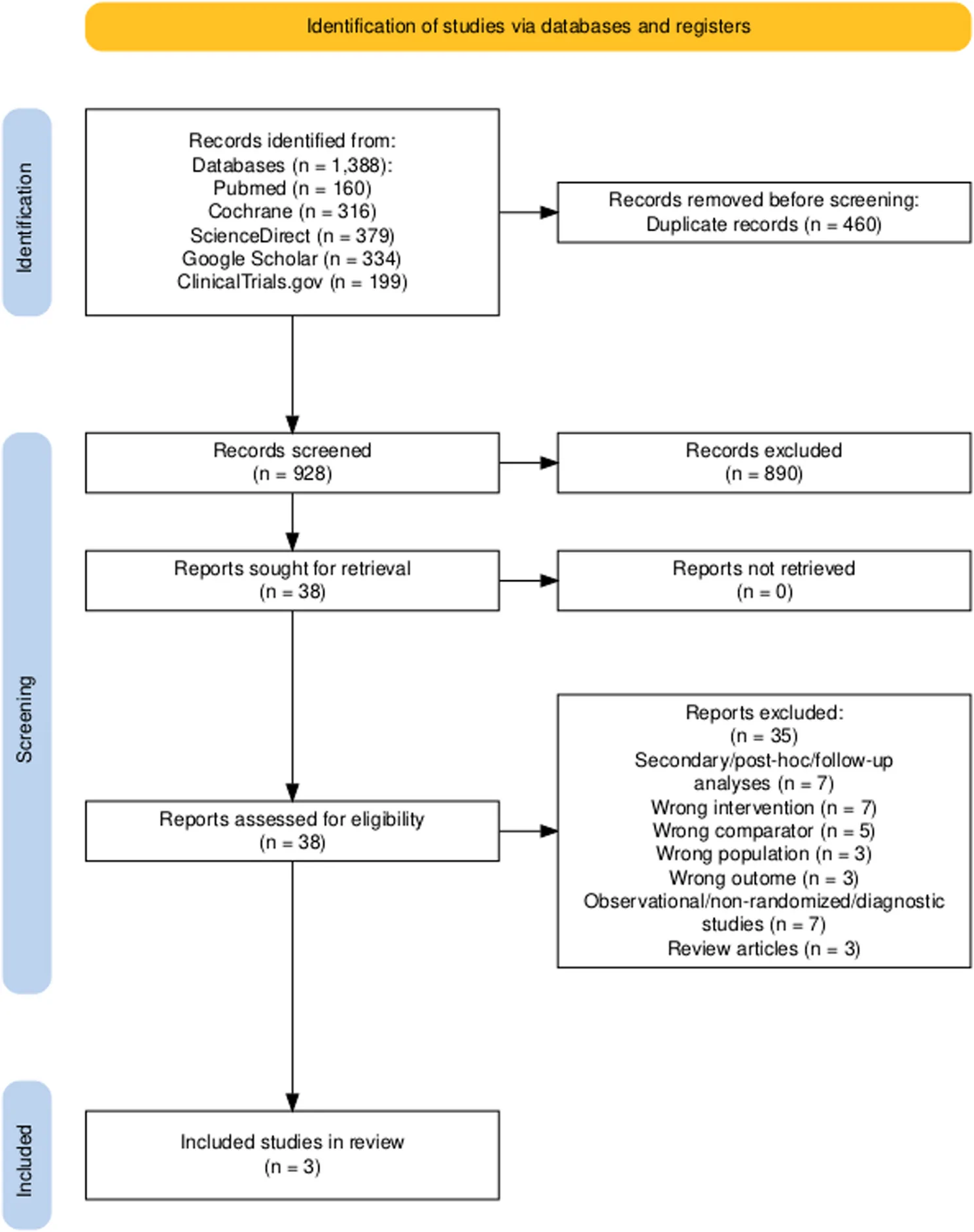
PRISMA flowchart showing the screening and study selection process

A total of 1,388 records were identified through database searches, including PubMed, Cochrane (CENTRAL), ScienceDirect, and Google Scholar. After removing 460 duplicate records, 928 studies remained for title and abstract screening. Of these, 890 records were excluded, and 38 reports were sought for full-text retrieval. All 38 articles were successfully retrieved and assessed for eligibility. Following full-text review, 35 studies were excluded. Ultimately, 3 RCTs met the inclusion criteria and were included in the final systematic review and meta-analysis. The study selection process is summarised in the PRISMA flow diagram (Fig. 1).

### Study characteristics

A total of 3 RCTs [8–10] comprising 6,165 patients were included in this meta-analysis. Clinical outcome analyses were performed using the analysed populations reported in the original studies (total *n* = 6,141), whereas procedural outcomes were pooled using the number of participants with available data for each respective endpoint. Supplementary Table S5 summarises the denominator used for procedural outcomes to improve transparency.

Follow-up duration was 1 year across all trials, with assessments at 30 days, 6 months, and/or 12 months. The mean age of participants ranged from 66.5 to 68.4 years. The included studies evaluated angiography-derived physiological assessment methods, including FFRangio, vFFR, and QFR. The comparator across all trials was pressure wire–based assessment using FFR or nonhyperaemic indices (e.g., iFR), with adenosine use as per protocol. The proportion of female participants ranged from 23.1% to 25%. The prevalence of diabetes mellitus ranged from 25.1% to 37.9%, while hypertension and dyslipidemia were reported in approximately 71% to 79% of patients. Detailed study characteristics are provided in Table 1.

**Table 1:** Trial-level summary of included studies

Variable	Fearon et al. (ALL-RISE)	Daemen et al. (FAST III)	Andersen al. (FAVOR III Europe)
Study design	International, randomised, noninferiority	International, randomized, open-label, controlled, noninferiority	Multicentre, randomised, open-label, noninferiority
Follow-up duration	1 year (30 days, 6 months, 12 months)	1 year (30 days, 12 months)	1 year (1 month, 12 months)
Total randomised, N (intervention/control)	1,930 (965/965)	2,235 (1124/1111)	2,000 (1008/992)
Intervention arm	FFRangio	vFFR	QFR
Control arm	Pressure wire–based assessment (FFR or [NHPR/iFR] with any commercially available wire)	Pressure wire–based assessment (FFR or [NHPR/iFR] with any commercially available wire)	Pressure wire–based FFR (standard clinical recommendations; adenosine; resting indices if adenosine intolerance)
Mean age ± SD, years	68.4 ± 10.2	67.4 ± 9.8	66.5 ± 10.3
Female sex, n (%)	482 (25)	538 (24.3)	462 (23.1)
Mean BMI ± SD, kg/m²	28.2 ± 5.75	27.7 ± 4.75	27.6 ± 4.4
Diabetes mellitus, n (%)	730/1,926 (37.9)	589/2,211 (26.6)	501/1,992 (25.1)
Hypertension, n (%)	1,526/1,926 (79.2)	1,602/2,211 (72.5)	1,529/1,993 (76.7)
Dyslipidaemia, n (%)	1,523/1,926 (79.1)	1,577/2,211 (71.3)	1,427/1,994 (71.6)
Current smoker, n (%)	274/1,926 (14.2)	416/2,211 (18.8)	487/1,900 (25.6)
Previous MI, n (%)	324/1,926 (16.8)	515/2,211 (23.3)	NR
Previous PCI, n (%)	750/1,926 (38.9)	771/2,211 (34.9)	977/1,994 (48.9)
Previous CABG, n (%)	*NR*	17/2,211 (0.8)	17/1,994 (0.9)

*FFR* Fractional flow reserve, *FFRangio* Angiography-derived FFR (CathWorks), *vFFR* vessel fractional flow reserve (Pie Medical Imaging), *QFR* Quantitative flow ratio (Medis), *NHPR* Nonhyperemic pressure ratio, *iFR* Instantaneous wave-free ratio, *SD* Standard deviation, *BMI*: body mass index, *MI*  Myocardial infarction, *PCI* Percutaneous coronary intervention, *CABG* Coronary artery bypass grafting

### Risk of bias assessment

**Fig. 2:**
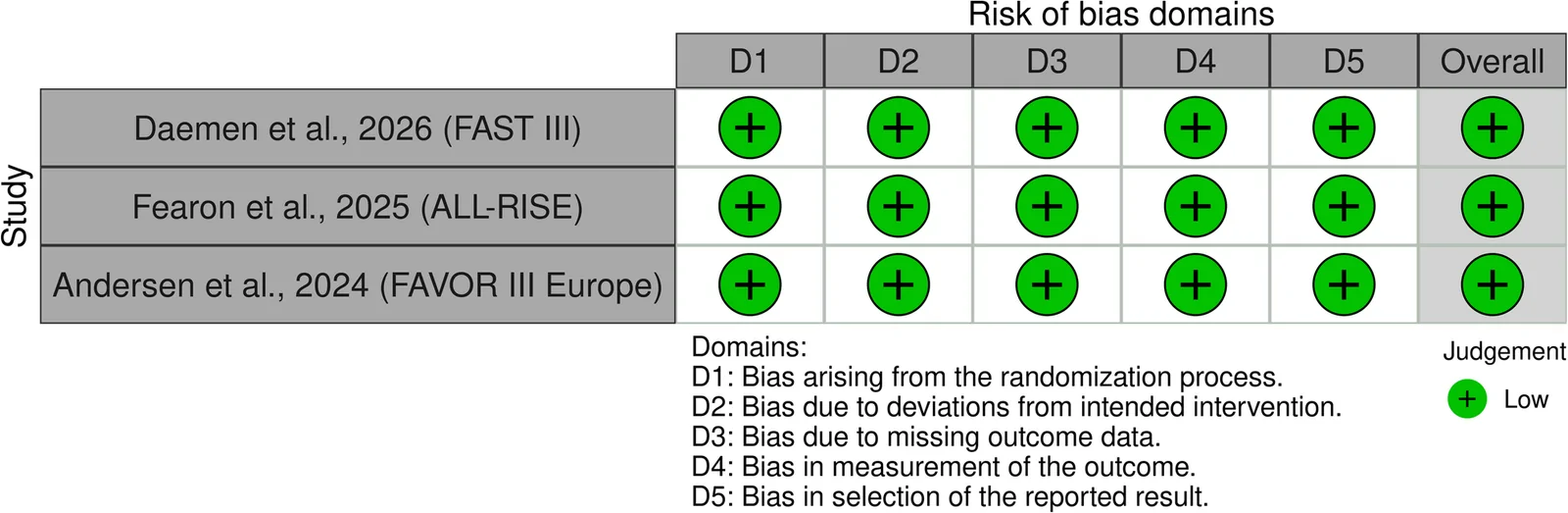
Risk of bias summary for the included trials

**Fig. 3:**
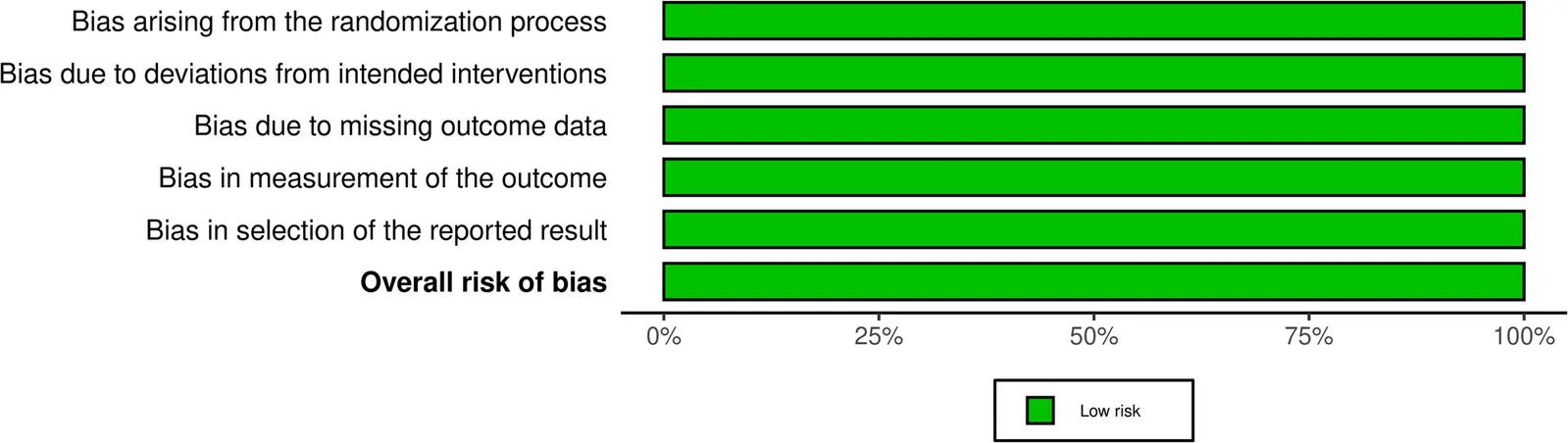
Risk of bias graph for the included trials

All included randomised controlled trials were assessed as having an overall low risk of bias across all five domains of the RoB 2.0 tool. Specifically, all studies demonstrated low risk of bias in the randomization process (D1), deviations from intended interventions (D2), missing outcome data (D3), measurement of outcomes (D4), and selection of the reported results (D5). Although all trials were open-label procedural studies, the risk of bias due to deviations from intended interventions was judged to be low because treatment allocation was randomised, interventions were protocol-driven, and the primary outcomes were objective clinical endpoints adjudicated by independent clinical events committees. No domain was judged to have “some concerns” or “high risk” in any of the included trials. Consequently, the overall risk of bias for all studies was classified as low (Figs. 2 and 3).

### GRADE assessment

The certainty of evidence was assessed using the GRADE framework. Overall, the certainty of evidence ranged from low to high. High-certainty evidence supported the outcomes of cardiac mortality, revascularization, total procedural time, and total fluoroscopy time. The certainty of evidence was rated as moderate for the primary composite endpoint and all-cause mortality, primarily because of inconsistency and imprecision, respectively. Evidence for MI was rated as low certainty owing to substantial heterogeneity and imprecision. Detailed GRADE assessments are presented in Supplementary Table S4.

## Results of meta-analysis

### Primary outcomes

#### Primary composite endpoint

**Fig. 4:**
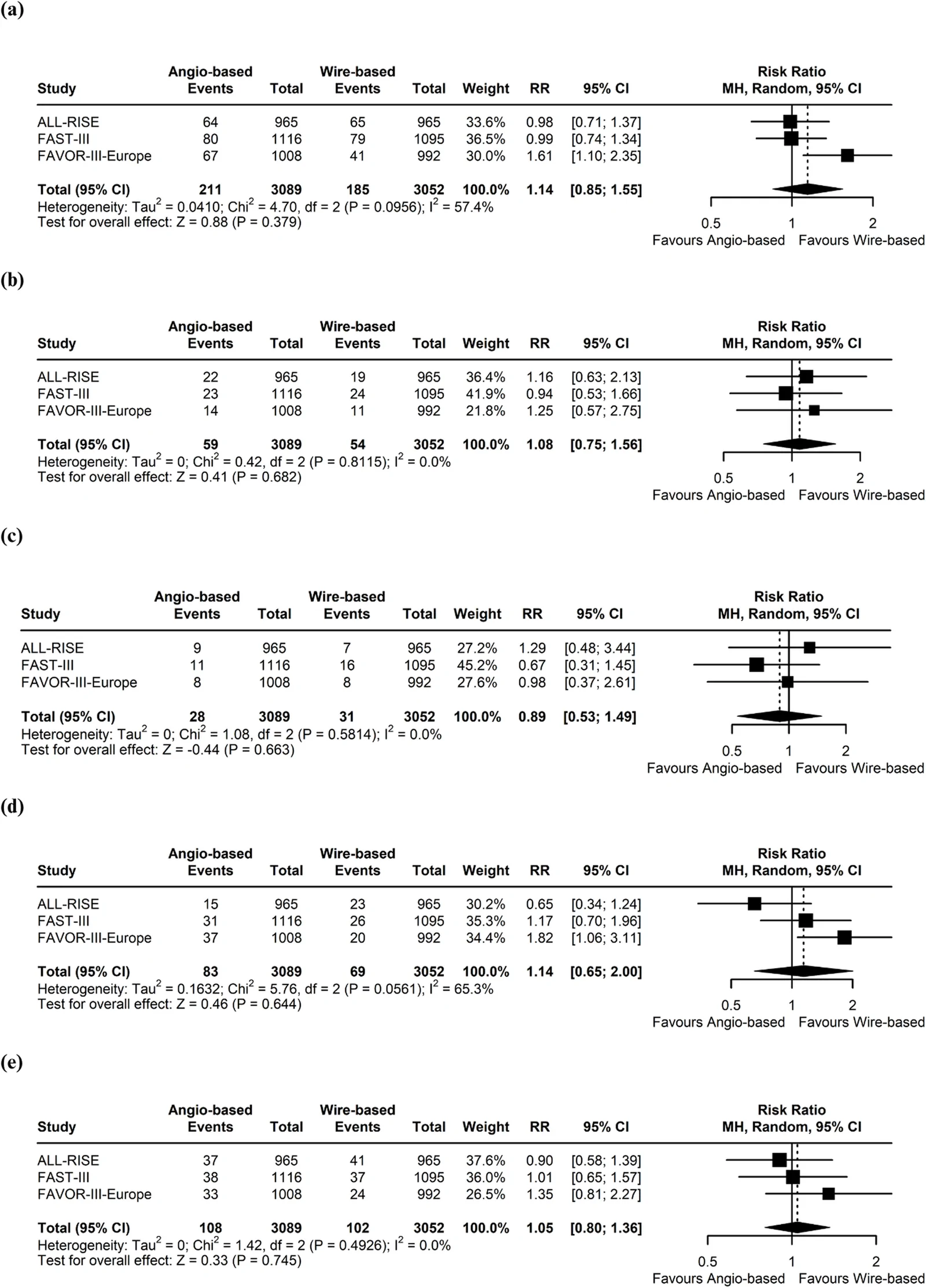
Forest plots of the primary clinical outcomes comparing angiography-based versus wire-based physiological assessment. **a** Primary composite outcome, (**b**) all-cause mortality, (**c**) cardiac mortality, (**d**) any myocardial infarction, and (**e**) revascularization. Effect estimates are presented as risk ratios (RRs) with 95% confidence intervals (CIs) using a random-effects (REML) model. Values < 1 favor angiography-based assessment, whereas values > 1 favor wire-based assessment

Three RCTs with a total of 6,141 patients reported primary composite. Meta-analysis demonstrated no significant difference between angiography-based and wire-based strategies (RR: 1.14; 95% CI: 0.85 to 1.55; *p* = 0.379; I² = 57.4%; Fig. 4a). Leave-one-out sensitivity analysis revealed that exclusion of the FAVOR-III-Europe trial (Andersen et al.) reduced heterogeneity to I² = 0% and changed the pooled estimate to (RR: 0.99; 95% CI: 0.79 to 1.24, Fig S9), without altering the overall conclusion. The DOI plot revealed an LFK index of 1.89, indicating minor asymmetry and a possible minor publication bias, as shown in Fig. S1.

#### All-cause mortality

Meta-analysis of three studies including 6,141 patients demonstrated no significant difference for all-cause mortality between angiography-based and wire-based strategies (RR: 1.08; 95% CI: 0.75 to 1.56; *p* = 0.682; I² = 0%; Fig. 4b). Leave-one-out sensitivity analysis demonstrated that sequential exclusion of each study did not materially alter the pooled effect estimate or the overall conclusion. (Fig S10). The DOI plot revealed an LFK index of − 0.21, indicating no major asymmetry and a low likelihood of publication bias, as shown in Fig S2.

#### Cardiac mortality

The risk of cardiac mortality outcome was reported in three randomised studies with a total of 6,141 participants. Pooled analysis showed no significant difference between the two groups (RR: 0.89; 95% CI: 0.53 to 1.49; *p* = 0.663; I² = 0%; Fig. 4c). Leave-one-out sensitivity analysis demonstrated that sequential exclusion of each study did not materially alter the pooled effect estimate or the overall conclusion. (Fig S11). The DOI plot revealed an LFK index of 0.20, indicating no major asymmetry and a low likelihood of publication bias, as shown in Fig S3.

#### Any myocardial infarction

The pooled analysis of three randomised trials, including 6,141 participants, demonstrated no significant difference in the risk for any MI between angiography-based and wire-based strategies (RR: 1.14; 95% CI: 0.65 to 2.00; *p* = 0.644; I² = 65.3%; Fig. 4d). Excluding ALL-RISE (Fearon et al.) reduced heterogeneity to I² = 26.4% while preserving the effect size (RR: 1.45; 95% CI: 0.94 to 2.24, Fig S12). The DOI plot revealed an LFK index of − 0.56, indicating no major asymmetry and a low likelihood of publication bias, as shown in Fig S4.

#### Revascularization

Three studies, including a total of 6,141 patients, assessed revascularization. Meta-analysis showed no significant difference between angiography-based and wire-based strategies (RR: 1.05; 95% CI: 0.80 to 1.36; *p* = 0.745; I² = 0%; Fig. 4e). Leave-one-out sensitivity analysis demonstrated that sequential exclusion of each study did not materially alter the pooled effect estimate or the overall conclusion. (Fig S13). The DOI plot revealed an LFK index of 1.05, indicating minor asymmetry and a possible minor publication bias, as shown in Fig S5.

### Secondary outcomes

#### Total procedural time

**Fig. 5:**
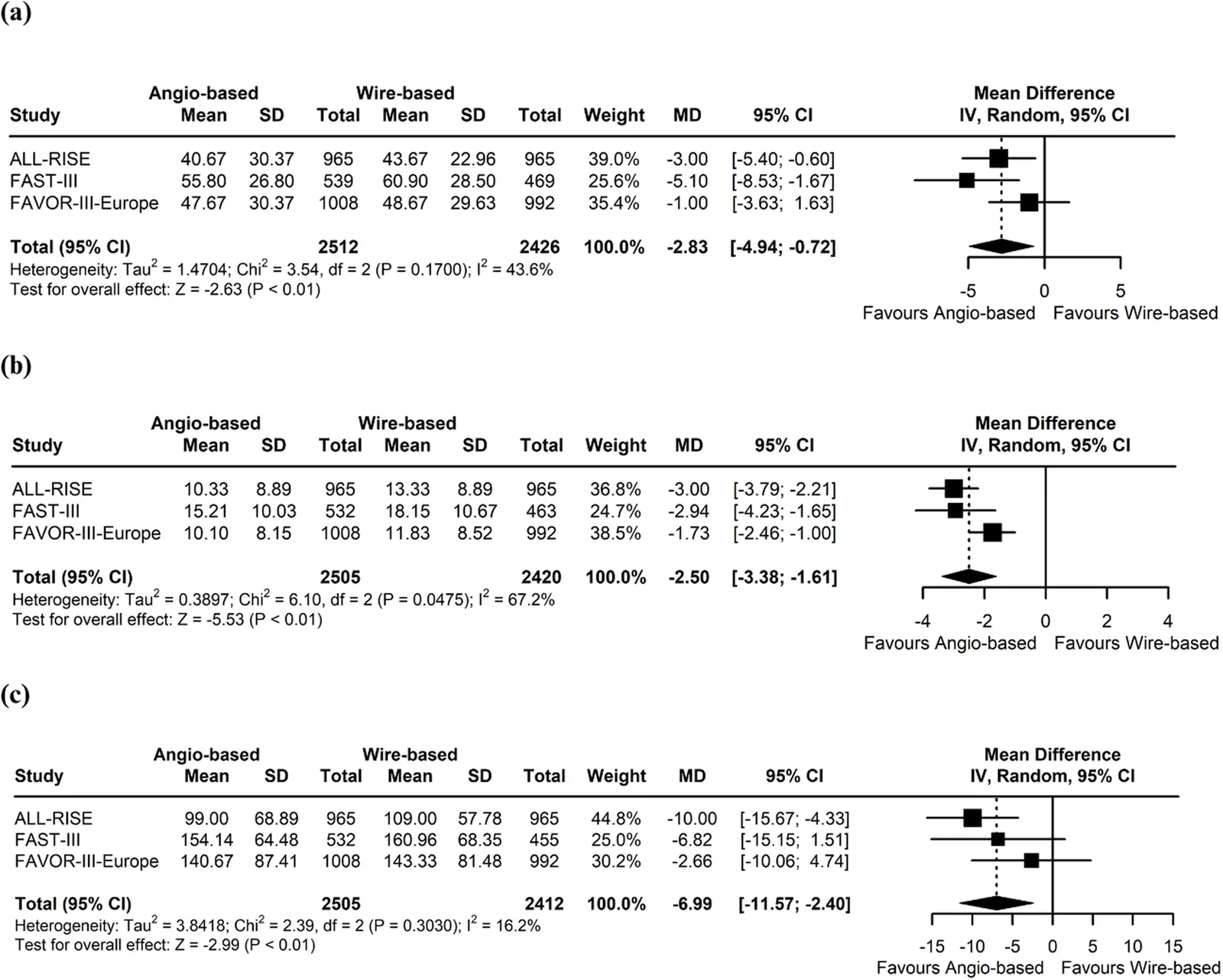
Forest plots of the secondary procedural outcomes comparing angiography-based versus wire-based physiological assessment. **a** Total procedural time, (**b**) total fluoroscopy time, and (**c**) contrast media volume. Effect estimates are presented as mean differences (MDs) with 95% confidence intervals (CIs) using a random-effects (REML) model. Negative values favor angiography-based assessment, whereas positive values favor wire-based assessment

Total procedural time was reported in three randomised studies comprising 4,938 participants. Pooled analysis showed that angiography-based guidance significantly reduced total procedural time compared with wire-based guidance (MD: − 2.83 min; 95% CI: − 4.94 to − 0.72; *p* < 0.01; I² = 43.6%; Fig. 5a). Leave-one-out sensitivity analysis indicated that omitting FAVOR-III-Europe (Andersen et al.) reduced heterogeneity to I² = 0%, while the pooled estimate remained in favor of angiography-based guidance (MD: − 3.69 min; 95% CI: − 5.66 to − 1.72; Fig S14). The DOI plot revealed an LFK index of − 0.49, indicating no major asymmetry and a low likelihood of publication bias, as shown in Fig S6.

#### Total fluoroscopy time

Three randomised studies comprising 4,925 participants reported total fluoroscopy time. Pooled analysis demonstrated that angiography-based guidance significantly reduced total fluoroscopy time compared with wire-based guidance (MD: − 2.50 min; 95% CI: − 3.38 to − 1.61; *p* < 0.01; I² = 67.2%; Fig. 5b). Leave-one-out sensitivity analysis revealed that removing FAVOR-III-Europe (Andersen et al.) reduced heterogeneity to I² = 0%, without substantially altering the effect size (MD: − 2.98 min; 95% CI: − 3.66 to − 2.31; Fig S15). The DOI plot revealed an LFK index of 1.02, indicating minor asymmetry and a possible minor publication bias, as shown in Fig S7.

#### Volume of contrast media

The meta-analysis of three randomised studies comprising 4,917 participants demonstrated that angiography-based guidance significantly reduced contrast volume compared with wire-based guidance (MD: − 6.99 mL; 95% CI: − 11.57 to − 2.40; *p* < 0.01; I² = 16.2%; Fig. 5c]. Leave-one-out sensitivity analysis revealed that removing either ALL-RISE (Fearon et al.) or FAVOR-III-Europe (Andersen et al.) reduced heterogeneity to I² = 0% without substantially altering the effect size (Fig S16). The DOI plot revealed an LFK index of 0.71, indicating no major asymmetry and a low likelihood of publication bias, as shown in Fig S8.

#### Subgroup analysis by type of comparator

A subgroup analysis was performed to examine the influence of the type of comparator used on different outcomes. Studies were divided into two groups: those using FFR alone (FAST-III and FAVOR-III-Europe), and those using a combination of FFR and NHPR (ALL-RISE). The interaction test revealed no statistically significant difference for primary composite, all-cause mortality, cardiac mortality, revascularization, total procedure time, total fluoroscopy time, and volume of contrast media, suggesting that the choice of comparator did not meaningfully alter the relative effect of angiography-based versus wire-based guidance (Fig S17-S23). However, a statistically significant subgroup interaction was observed for any MI, indicating that the comparator type modified the treatment effect between angiography-based and wire-based guidance (Fig S24). However, given the inclusion of only three trials, these subgroup findings should be interpreted as exploratory and hypothesis-generating.

#### Subgroup analysis by type of technology

A subgroup analysis was performed based on the type of technology used. Studies were divided into two groups: those using patient-specific hemodynamic systems (ALL-RISE and FAST-III), and those using fixed-flow model-based systems (FAVOR-III-Europe). The interaction test revealed no statistically significant difference for all-cause mortality, cardiac mortality, any MI, revascularization, total procedure time, and volume of contrast media, indicating that the type of technology did not significantly modify the comparative effectiveness of angiography-based versus wire-based strategies (Fig S25-S30). In contrast, a statistically significant subgroup interaction was observed for the primary composite outcome and total fluoroscopy time (Fig. S31 and S32). However, these findings should be interpreted cautiously because the subgroup analysis was based on only three trials and was not powered to establish definitive platform-specific effects.

## Discussion

Across three randomised trials evaluating angiography-derived physiology against pressure-wire-based physiology, this meta-analysis suggests that angiography-derived strategies achieve broadly comparable 1-year clinical outcomes while offering important procedural efficiencies. No statistically significant difference was observed for the primary composite endpoint, all-cause mortality, cardiac mortality, any MI, or revascularization. By contrast, angiography-derived guidance significantly reduced total procedural time, fluoroscopy time, and contrast use. The principal advantage of angiography-derived physiology, therefore, appears to reside not in producing superior hard clinical outcomes beyond current wire-based standards, but in simplifying invasive decision-making while preserving comparable overall clinical effectiveness.

These findings must be interpreted against the established position of wire-based physiology as the reference standard for the functional assessment of intermediate coronary lesions. FFR-guided revascularization became the accepted benchmark after landmark trials such as FAME and FAME 2 demonstrated the superiority of physiology-guided decision-making over angiographic assessment alone and confirmed the long-term clinical validity of pressure-wire-based lesion selection [2, 3, 15]. Current guideline frameworks are built on that evidence base, which explains why newer angiography-derived approaches are being judged not against angiography alone, but against a comparator that already has strong trial and guideline support [4, 16].

At the same time, the practical limitations of wire-based physiology have long been recognized. Pressure-wire manipulation, the use of hyperaemic agents in FFR-based workflows, additional cost, longer procedure duration, and patient discomfort all contribute to the underuse of physiology in routine practice. The development of NHPR such as iFR was itself driven by the need to simplify physiology-guided revascularization without sacrificing clinical validity, and both DEFINE-FLAIR and iFR-SWEDEHEART showed that less burdensome physiological strategies could preserve outcomes while improving procedural efficiency [17, 18]. In this context, Angiography-derived physiology should be viewed as a further step along the same trajectory: an attempt to preserve the benefits of physiology-guided care while removing some of the practical barriers that limit adoption.

The procedural findings of the present meta-analysis are therefore clinically coherent. Angiography-derived physiology reduced procedure time, fluoroscopy exposure, and contrast volume, which are among the most tangible and immediately relevant advantages of a wire-free strategy. Mechanistically, this is plausible because these platforms avoid routine pressure-wire crossing, reduce or eliminate dependence on hyperemia, and may shorten the interval between angiographic acquisition and treatment decision. This interpretation is also consistent with recent expert and review literature describing angiography-derived physiology as an approach that may facilitate wider adoption of physiology-guided practice by reducing procedural complexity and workflow burden [19, 20].

However, the most important interpretive point in this review is that angiography-derived physiology should not be treated as a single uniform intervention. The three included randomised trials assessed distinct technological platforms with different computational assumptions and workflow requirements: ALL-RISE (Fearon et al.) evaluated FFRangio using software-assisted resistance modeling, FAST III (Daemen et al.) assessed vFFR using angiographic reconstruction and 3D quantitative coronary angiography, and FAVOR III Europe (Andersen et al.) evaluated QFR using a contrast-flow-based approach. Contemporary mechanistic and state-of-the-art reviews have emphasized that these systems differ in their reconstruction methods, assumptions regarding flow and resistance, boundary-condition modeling, and software architecture, meaning that they are not simply interchangeable versions of the same physiological test [21–23]. Accordingly, the pooled estimate for the primary composite endpoint should be interpreted with appropriate caution, given the heterogeneity in both angiography-derived platforms and trial-specific composite endpoint definitions. This distinction is not merely technical; it has the potential to affect lesion classification, operator confidence, treatment deferral patterns, and ultimately clinical outcomes.

This platform-specific perspective is supported by the exploratory subgroup analyses of the present study. A significant interaction by technology type was observed for the primary composite outcome and total fluoroscopy time, suggesting that the comparative effect of angiography-derived physiology may depend in part on the underlying computational platform. This observation carries substantive implications for how pooled evidence across heterogeneous platforms should be interpreted: it argues against assuming a simple class effect across all angiography-derived systems and suggests instead that algorithm design, software workflow, and physiological modeling may modify both procedural and clinical performance. Recent consensus and review papers have similarly emphasized that platform-specific differences remain a central issue in the evaluation of angiography-derived physiology and that individual systems should not automatically be regarded as equivalent solely because they are all wire-free [19–22].

The significance of the technology interaction for the primary composite outcome deserves particular emphasis. The absence of significant subgroup interaction across each component outcome does not negate this observation. Composite endpoints generally provide greater statistical power than their individual components, especially in a meta-analysis based on only three randomised trials and relatively few events per endpoint. It is therefore plausible that a true technology-dependent pattern would become detectable first at the composite level before becoming evident for less frequent component outcomes. In that sense, the current findings do not prove the superiority of one platform over another, but they do support the conclusion that clinically relevant platform-level differences may exist and deserve focused investigation in future trials.

The divergent external evidence surrounding the Quantitative Flow Ratio (QFR) further strengthens this interpretation. In FAVOR III China, QFR-guided PCI improved outcomes compared with angiography-guided PCI, supporting the concept that angiography-derived physiology can outperform anatomy-based decision-making [24]. By contrast, FAVOR III Europe did not demonstrate noninferiority of QFR versus FFR when a pressure-wire reference standard was available, and a subsequent post hoc analysis showed that QFR-based deferral was associated with a higher 1-year risk of major adverse cardiovascular events than FFR-based deferral [25]. That contrast is highly informative. It suggests that the favorable performance of one angiography-derived platform in one comparator setting should not be generalized uncritically across all platforms or all clinical contexts. Rather than weakening the field as a whole, these divergent findings reinforce the need for platform-aware interpretation.

Comparator-based subgroup analyses in the present review are useful but less compelling mechanistically than the technology subgroup. FAST III and FAVOR III Europe used pure FFR comparators, whereas ALL-RISE employed a mixed pressure-wire comparator that included both FFR and NHPR. This matters because hyperaemic and non-hyperaemic indices are not identical reference standards, and differences in comparator composition may influence apparent treatment effects. At the same time, the mixed comparator subgroup is represented by only one trial, which limits formal inference and makes it difficult to separate comparator-related effects from trial-specific and technology-specific influences. Thus, although comparator heterogeneity should be acknowledged, it does not diminish the conceptual importance of the technology subgroup; if anything, it reinforces the need to interpret these trials at the level of both platform and comparator design.

The heterogeneity seen in some pooled analyses should also be considered in this framework. Moderate to substantial heterogeneity was observed for outcomes such as the primary composite and any MI, with sensitivity analyses indicating that the observed heterogeneity was largely driven by the FAVOR III Europe trial. Exclusion of this study substantially reduced heterogeneity without changing the overall direction of the pooled estimates, suggesting that the apparent variability reflects trial-specific characteristics rather than contradictory treatment effects. This pattern suggests variation in effect magnitude rather than true contradiction. Given the differences among angiography-derived platforms and the evolving nature of this technology class, such variability is not unexpected. Recent reviews likewise describe a field in transition: angiography-derived physiology has shown strong diagnostic correlation with invasive physiology, but clinical-outcome evidence remains comparatively limited, and broader implementation still depends on a clearer understanding of which platforms perform best, in which settings, and under what assumptions [20–22].

These considerations also have guideline implications. Current European guidance has incorporated angiography-derived physiology into the chronic coronary syndrome (CCS) framework, reflecting growing confidence in wire-free physiological assessment [16]. Yet the broader revascularization framework remains fundamentally grounded in the established evidence supporting FFR- and NHPR-guided lesion assessment [4]. The present findings fit squarely within that tension. On the one hand, angiography-derived physiology appears capable of preserving clinical effectiveness while improving efficiency. On the other hand, the technology-specific subgroup findings and divergent trial-level behavior caution against treating all wire-free platforms as equivalent substitutes for pressure-wire physiology in every clinical setting.

### Limitations

Several limitations should be acknowledged. First, only three randomised trials were included, which restricts precision and limits the power of subgroup analyses. Additionally, given the small number of included studies, confidence intervals may be slightly anti-conservative despite the use of a REML random-effects model, and the findings should be interpreted cautiously. Second, while the technology subgroup findings are clinically plausible and statistically informative, they remain based on very small evidence base and should therefore be regarded as hypothesis-generating rather than definitive. Third, comparator-based subgroup analysis is inherently limited because the mixed FFR-plus-NHPR subgroup was represented by a single trial. Fourth, procedural trials of this kind are open-label by design and are therefore vulnerable to operator learning effects, workflow bias, and trial-specific implementation differences. Finally, one-year outcomes may not fully capture clinically important differences in deferred-lesion safety or platform-specific performance trajectories that may only become apparent with extended follow-up, a consideration of particular relevance given the divergent deferral safety signals observed across platforms in external analyses.

## Conclusion

In summary, the available randomised evidence suggests that angiography-derived physiology can achieve similar 1-year clinical outcomes to pressure-wire-based physiology while offering meaningful reductions in procedural time, fluoroscopy exposure, and contrast use. The most important insight from this meta-analysis, however, is that angiography-derived physiology should not be interpreted as a uniform class. The significant interaction by technology type for the primary composite outcome and fluoroscopy time, together with divergent findings across external platform-specific studies, suggests that algorithm design, software architecture, and computational physiology may materially influence performance. Future research should therefore move beyond the broad question of whether angiography-derived physiology works in general and instead determine which platforms work best, in which lesion subsets, and under which clinical conditions.

## Supplementary Information

Supplementary Material 1.

## Data Availability

The data that support the findings of this study are available from the corresponding author upon reasonable request.

## Abbreviations

FFR: Fractional Flow Reserve
PCI: Percutaneous Coronary Intervention
RR: Risk Ratio
MD: Mean Differences
CI: Confidence Interval
MI: Myocardial Infarction
IHD: Ischemic heart disease
PRISMA: Preferred Reporting Items for Systematic Reviews and Meta-Analyses
CENTRAL: Cochrane Central Register of Controlled Trials
RCTs: Randomised Controlled Trials
REML: Restricted Maximum Likelihood
NHPR: Non-Hyperaemic Pressure Ratios
QFR: Quantitative Flow Ratio
CCS: Chronic Coronary Syndrome
Rob 2.0: Revised Cochrane Risk-of-Bias tool for randomised trials
GRADE: Grading of Recommendations, Assessment, Development, and Evaluations
