# Supplementary material for: Angiography-derived versus pressure-wire–based physiological assessment to guide percutaneous coronary intervention: a systematic review and meta-analysis of randomised controlled trials
Source: BMC Cardiovasc Disord. 2026 Jul 18;26:915. doi: 10.1186/s12872-026-06305-7 (PMC13663117; doi:10.1186/s12872-026-06305-7)
Supplement: Supplementary file 1 [file 12872_2026_6305_MOESM1_ESM.docx]

Online supplementary Table 1: Search Strategy

| Database | Search string | Results |
| --- | --- | --- |
| Pubmed | ("angiography derived" OR "angiography‑derived" OR "angiography derived FFR" OR "FFRangio" OR "quantitative flow ratio" OR "QFR" OR "vessel FFR" OR "Angio‑FR" OR "image derived FFR") AND ("pressure‑wire" OR "pressure wire" OR fractional flow reserve OR FFR OR "nonhyperemic pressure ratio" OR NHPR OR iFR) AND (percutaneous coronary intervention OR PCI OR revascularization) AND ("coronary angiography" OR coronary stenosis) AND ("randomized" OR trial) | 160 |
| Google Scholar | ("quantitative flow ratio" OR QFR) AND ("fractional flow reserve") AND ("percutaneous coronary intervention") AND ("randomized controlled trial") | 334 |
| Cochrane | #1 "angiography-derived" OR "FFRangio" OR QFR OR "quantitative flow ratio" :ti,ab #2 "pressure-wire" OR FFR OR iFR OR "fractional flow reserve" :ti,ab #3 "percutaneous coronary intervention" OR PCI OR "coronary intervention" :ti,ab #4 "death" OR "myocardial infarction" OR "MI" OR "major adverse cardiac events" OR MACE :ti,ab #5 "randomized controlled trial" OR RCT:pt #6 #1 AND #2 AND #3 AND #4 AND #5 | 316 |
| ScienceDirect | ("quantitative flow ratio" OR QFR) AND ("fractional flow reserve") AND ("percutaneous coronary intervention") AND (randomized OR trial) | 379 |
| Clinicaltrial.gov | ("angiography" OR "coronary imaging" OR "coronary physiology" OR "functional assessment" OR "physiologic assessment") AND ("percutaneous coronary intervention" OR PCI OR "coronary intervention" OR "coronary revascularization") | 199 |

# Supplementary Table S2. Full-text articles excluded after eligibility assessment (n = 35)

| # | First author | Year | DOI | Primary reason for exclusion |
| --- | --- | --- | --- | --- |
| 1 | Hu | 2025 | 10.1016/S0140-6736(25)00504-5 | Wrong comparator (angiography-derived FFR vs IVUS) |
| 2 | Erriquez | 2024 | 10.1016/j.jcin.2024.04.022 | Post-hoc analysis of an included RCT |
| 3 | Guo | 2024 | 10.1148/radiol.233354 | Wrong intervention (CT-derived FFR) |
| 4 | Asano | 2024 | 10.4244/EIJ-D-23-00674 | Wrong outcome |
| 5 | Biscaglia (AQVA-II) | 2024 | 10.1016/j.jcin.2023.10.032 | Wrong intervention |
| 6 | Dai | 2025 | 10.1161/JAHA.124.035756 | Secondary analysis of included trial |
| 7 | Fargione | 2026 | 10.3390/medicina62020313 | Narrative Review |
| 8 | Biscaglia (AQVA) | 2023 | 10.1016/j.jcin.2022.10.054 | Wrong comparator |
| 9 | Lee | 2023 | 10.1016/j.jcin.2023.08.032 | Observational/post-hoc analysis |
| 10 | Zheng | 2025 | 10.1016/j.jcin.2024.10.017 | Wrong outcome |
| 11 | Kwon | 2024 | 10.1161/CIRCINTERVENTIONS.123.013844 | Diagnostic substudy |
| 12 | D'Ascenzo | 2025 | 10.1136/openhrt-2025-003453 | Wrong comparator |
| 13 | Zhao | 2025 | 10.1097/CM9.0000000000003484 | Economic/secondary analysis of included trial |
| 14 | Peper | 2023 | 10.1007/s11596-023-2751-4 | Narrative Review |
| 15 | Zhang | 2022 | 10.4244/EIJ-D-21-00176 | Non-randomized study |
| 16 | Zhang | 2025 | 10.1016/j.jcin.2024.09.045 | Secondary analysis |
| 17 | Barauskas | 2023 | 10.1186/s12872-023-03153-7 | Wrong comparator |
| 18 | Qian | 2025 | 10.1093/bjr/tqaf055 | Wrong intervention (CT-derived FFR) |
| 19 | Zhang | 2026 | 10.1016/j.jcin.2025.10.042 | Post-hoc analysis |
| 20 | Erbay | 2024 | 10.1016/j.ahj.2024.07.011 | Observational study |
| 21 | Chen | 2024 | 10.1161/CIRCINTERVENTIONS.124.014362 | Wrong outcome |
| 22 | Andersen | 2025 | 10.4244/EIJ-D-24-01001 | Post-hoc analysis of included trial |
| 23 | Cai | 2021 | 10.1002/ccd.29640 | Wrong population |
| 24 | Zhu | 2026 | 10.1016/S0140-6736(25)02418-3 | Wrong population |
| 25 | Hoek | 2025 | 10.1161/CIRCIMAGING.125.018574 | Diagnostic study |
| 26 | Lin | 2024 | 10.1186/s12872-024-04413-w | Wrong population |
| 27 | Aurigemma | 2024 | 10.1161/CIRCINTERVENTIONS.123.013191 | Long-term follow-up of previous trial |
| 28 | Nørgaard | 2014 | 10.1016/j.jacc.2013.11.043 | Diagnostic study (CT-derived FFR) |
| 29 | Andreini | 2019 | 10.1161/CIRCINTERVENTIONS.118.007607 | Wrong intervention (CT-derived FFR) |
| 30 | Ullrich-Daub | 2025 | 10.1007/s00392-024-02484-5 | Wrong comparator |
| 31 | Bär | 2021 | 10.1161/JAHA.120.019052 | Observational study |
| 32 | Gabara | 2025 | 10.1161/JAHA.123.029950 | Wrong intervention (CT-derived FFR substudy) |
| 33 | Park | 2015 | 10.1161/CIRCINTERVENTIONS.115.002442 | Wrong intervention |
| 34 | Huang | 2020 | 10.1177/0846537119893752 | Review article |
| 35 | Wykrzykowska | 2012 | 10.4244/EIJV8I8A144 | Wrong intervention/device study |

Supplementary Table S3 **Primary Composite Endpoint**

| **Trial** | **Definitions** |
| --- | --- |
| ALL-RISE | Composite: all-cause death + MI + unplanned clinically indicated revascularization |
| FAST-III | Composite: all-cause death + MI + ANY revascularization (not restricted to unplanned) |
| FAVOR-III-Europe | Composite: all-cause death + MI + unplanned coronary revascularization |

| **Supplementary Table S4 Certainty of Evidence** | | | | | | | | | | | |
| --- | --- | --- | --- | --- | --- | --- | --- | --- | --- | --- | --- |
| **Certainty assessment** | | | | | | | **Summary of findings** | | | | |
| **Participants (studies) Follow-up** | **Risk of bias** | **Inconsistency** | **Indirectness** | **Imprecision** | **Publication bias** | **Overall certainty of evidence** | **Study event rates (%)** | | **Relative effect (95% CI)** | **Anticipated absolute effects** | |
|  |  |  |  |  |  |  | **With WDP** | **With ADP** |  | **Risk with WDP** | **Risk difference with ADP** |
| **Primary Composite Endpoint** | | | | | | | | | | | |
| 6141 (3 RCTs) | not serious | serious^a^ | not serious | not serious | none | ⨁⨁⨁◯ Moderate^a^ | 185/3052 (6.1%) | 211/3089 (6.8%) | **RR 1.14** (0.85 to 1.55) | 185/3052 (6.1%) | **8 more per 1,000** (from 9 fewer to 33 more) |
| **All-Cause Mortality** | | | | | | | | | | | |
| 6141 (3 RCTs) | not serious | not serious | not serious | serious^b^ | none | ⨁⨁⨁◯ Moderate^b^ | 54/3052 (1.8%) | 59/3089 (1.9%) | **RR 1.08** (0.75 to 1.56) | 54/3052 (1.8%) | **1 more per 1,000** (from 4 fewer to 10 more) |
| **Cardiac Mortality** | | | | | | | | | | | |
| 6141 (3 RCTs) | not serious | not serious | not serious | not serious | none | ⨁⨁⨁⨁ High | 31/3052 (1.0%) | 28/3089 (0.9%) | **RR 0.89** (0.53 to 1.49) | 31/3052 (1.0%) | **1 fewer per 1,000** (from 5 fewer to 5 more) |
| **Any Myocardial Infarction** | | | | | | | | | | | |
| 6141 (3 RCTs) | not serious | serious^c^ | not serious | serious^d^ | none | ⨁⨁◯◯ Low^c,d^ | 69/3052 (2.3%) | 83/3089 (2.7%) | **RR 1.14** (0.65 to 2.00) | 69/3052 (2.3%) | **3 more per 1,000** (from 8 fewer to 23 more) |
| **Revascularization** | | | | | | | | | | | |
| 6141 (3 RCTs) | not serious | not serious | not serious | not serious | none | ⨁⨁⨁⨁ High | 102/3052 (3.3%) | 108/3089 (3.5%) | **RR 1.05** (0.80 to 1.36) | 102/3052 (3.3%) | **2 more per 1,000** (from 7 fewer to 12 more) |
| **Total Procedural Time** | | | | | | | | | | | |
| 4938 (3 RCTs) | not serious | not serious | not serious | not serious | none | ⨁⨁⨁⨁ High | 2426 | 2512 | - | 2426 | MD **2.83 lower** (4.94 lower to 0.72 lower) |
| **Total Fluoroscopy Time** | | | | | | | | | | | |
| 4925 (3 RCTs) | not serious | not serious | not serious | not serious | none | ⨁⨁⨁⨁ High | 2420 | 2505 | - | 2420 | MD **2.5 lower** (3.38 lower to 1.61 lower) |

**CI:** confidence interval; **MD:** mean difference; **RR:** risk ratio

#### Explanations

a. I² = 57.4% represents moderate heterogeneity, and the sensitivity analysis showed heterogeneity was largely driven by one study (FAVOR III Europe).

b. Downgrade because mortality events are relatively infrequent and the confidence interval includes both a clinically important reduction and an increase in mortality.

c. Downgraded one level for inconsistency due to substantial heterogeneity across studies (I² = 65.3%)

d. Downgraded one level for imprecision because the 95% confidence interval was wide and included both clinically important benefit and harm

**Supplementary Table S5:** Total number of patients analyzed in procedural outcomes

| **Total Procedural Time** | | |
| --- | --- | --- |
| Trial | Intervention(n) | Control(n) |
| ALL-RISE | 965 | 965 |
| FAST-III | 539 | 469 |
| FAVOR-III-Europe | 1008 | 992 |
| **Total Fluoroscopy Time** | | |
| Trial | Intervention(n) | Control(n) |
| ALL-RISE | 965 | 965 |
| FAST-III | 532 | 463 |
| FAVOR-III-Europe | 1008 | 992 |
| **Volume of Contrast Media** | | |
| Trial | Intervention(n) | Control(n) |
| ALL-RISE | 965 | 965 |
| FAST-III | 539 | 455 |
| FAVOR-III-Europe | 1008 | 992 |

**Supplementary Fig S1** Primary Composite (Doi Plot and LFK index)

**Supplementary Fig S2** All-Cause Mortality (Doi Plot and LFK index)

**Supplementary Fig S3** Cardiac Mortality (Doi Plot and LFK index)

**Supplementary Fig S4** Any Myocardial Infarction (Doi Plot and LFK index)

**Supplementary Fig S5** Revascularization (Doi Plot and LFK index)

**Supplementary Fig S6** Total Procedural Time (Doi Plot and LFK index)

**Supplementary Fig S7** Total Fluoroscopy Time (Doi Plot and LFK index)

**Supplementary Fig S8** Volume of Contrast Media (Doi Plot and LFK index)

**Supplementary Fig S9** Primary Composite (Sensitivity Analysis)

**Supplementary Fig S10** All-Cause Mortality(Sensitivity Analysis)

**Supplementary Fig S11** Cardiac Mortality (Sensitivity Analysis)

**Supplementary Fig S12** Any Myocardial Infarction (Sensitivity Analysis)

**Supplementary Fig S13** Revascularization (Sensitivity Analysis)

**Supplementary Fig S14** Total Procedural Time (Sensitivity Analysis)

**Supplementary Fig S15** Total Fluoroscopy Time (Sensitivity Analysis)

**Supplementary Fig S16** Volume of Contrast Media (Sensitivity Analysis)

**SUBGROUP ANALYSIS BASED ON COMPARATOR** **Supplementary Fig S17** Primary Composite

**Supplementary Fig S18** All-Cause Mortality

**Supplementary Fig S19** Cardiac Mortality

**Supplementary Fig S20** Revascularization

**Supplementary Fig S21** Total Procedural Time

**Supplementary Fig S22** Total Fluoroscopy Time

**Supplementary Fig S23** Volume of Contrast Media

**Supplementary Fig S24** Any Myocardial Infarction


**SUBGROUP ANALYSIS BASED ON TECHNOLOGY** **Supplementary Fig S25** All-Cause Mortality

**Supplementary Fig S26** Cardiac Mortality

**Supplementary Fig S27** Any Myocardial Infarction

**Supplementary Fig S28** Revascularization

**Supplementary Fig S29** Total Procedural Time

**Supplementary Fig S30** Volume of Contrast Media

**Supplementary Fig S31** Primary Composite

**Supplementary Fig S32** Total Fluoroscopy Time
